# Integrated mRNA and miRNA transcriptomic analysis reveals the response of *Rapana venosa* to the metamorphic inducer (juvenile oysters)

**DOI:** 10.1016/j.csbj.2022.12.047

**Published:** 2022-12-30

**Authors:** Mei-Jie Yang, Hao Song, Pu Shi, Jian Liang, Zhi Hu, Cong Zhou, Peng-Peng Hu, Zheng-Lin Yu, Tao Zhang

**Affiliations:** a CAS Key Laboratory of Marine Ecology and Environmental Sciences, Institute of Oceanology, Chinese Academy of Sciences, Qingdao, China; b Laboratory for Marine Science and Technology, Qingdao National Laboratory for Marine Science and Technology, Qingdao, China; c Center for Ocean Mega-Science, Chinese Academy of Sciences, Qingdao, China; d CAS Engineering Laboratory for Marine Ranching, Institute of Oceanology, Chinese Academy of Sciences, Qingdao, China; e University of Chinese Academy of Sciences, Beijing 100049, China; f Shandong Province Key Laboratory of Experimental Marine Biology, Qingdao 266071, China; g Tianjin Key Laboratory of Aqua-ecology and Aquaculture, Fisheries College, Tianjin Agricultural University, Tianjin 300384, China; h Research and Development Center for Efficient Utilization of Coastal Bioresources, Yantai Institute of Coastal Zone Research, Chinese Academy of Sciences, Yantai 264003, China

**Keywords:** *Rapana venosa*, Metamorphic inducer, mRNA, miRNA

## Abstract

Metamorphosis, as a critical developmental event, controls the population dynamics of most marine invertebrates, especially some carnivorous gastropods that feed on bivalves, whose population dynamics not only affect the maintenance of the ecological balance but also impact the protection of bivalve resources; therefore, the metamorphosis of carnivorous gastropods deserve attention. Here, we investigated the mechanism underlying the response of the carnivorous gastropod *Rapana venosa* to its metamorphic inducer juvenile oysters through integrated analysis of miRNA and mRNA profiles. According to the results, we speculated that the AMPK signaling pathway may be the critical regulator in the response to juvenile oysters in *R. venosa* competent larvae. The NF-kB and JAK-STAT signaling pathways that regulated apoptosis were also activated by the metamorphic inducer, which may result in the degeneration of the velum. Additionally, the significant changes in the expression of the SARP-19 precursor gene and protein cibby homolog 1-like gene may indicate that these signaling pathways also regulate growth and development during metamorphosis. This study provides further evidence that juvenile oysters can induce metamorphosis of *R. venosa* at the transcriptional level, which expands our understanding of the metamorphosis mechanism in carnivorous gastropods*.*

## Graphical Abstract

**Figure:**
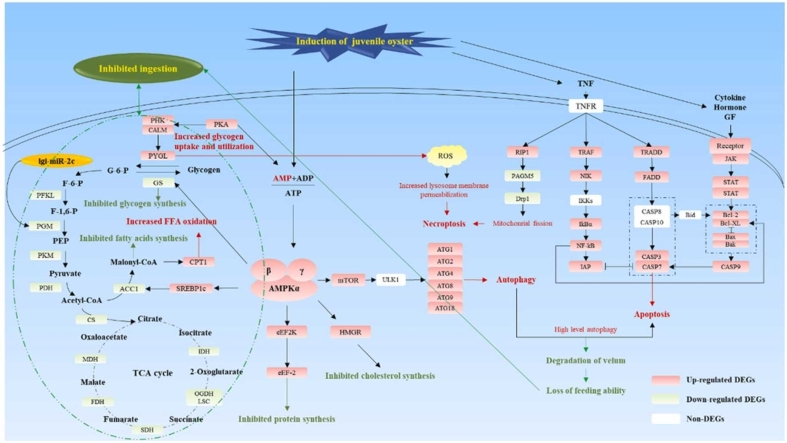

## 1 Introduction

Most marine invertebrates have biphasic life cycles, and metamorphosis is critical for the completion of their life cycle due to their sensitivity and vulnerability [1]. Therefore, metamorphosis controls the population dynamics of marine invertebrates. Metamorphosis occurs non-spontaneously in almost all marine invertebrates. Bacteria-stimulated metamorphosis is the most extensively researched and is widespread in almost all clades of marine invertebrates [2]. In addition, conspecific adults and food also stimulate metamorphosis in some species. Metamorphosis stimulated by conspecific adults also occurs in some fouling species, such as *Mytilopsis sallei* and *Balanus amphitrite* [3], [4]*.* Food-stimulated metamorphosis has been observed in some gastropods, such as the herbivorous gastropods *Haliotis rufescens* and *Crepidula fornicata*, whose metamorphosis is stimulated by their food species *Lithophyllum* sp. and *Lithothamnion* sp. [5], [6], and the carnivorous gastropod *Onchidoris bilamellata*, whose metamorphosis is induced by its prey, juvenile barnacles [7].

*Rapana venosa* is a typical carnivorous gastropod that feeds mainly on bivalves and has high edibility and medicinal value [8], [9], [10], [11]. Considering the importance of metamorphosis to animal population dynamics, we conducted a series of studies on the metamorphosis of *R. venosa*, including its morphology, behavior, enzyme kinetics, symbiotic microbiota, metabolome, proteome, transcriptome and critical genes [12], [13], [14], [15], [16], [17], [18]. Interestingly, we found that juvenile oysters (shell length less than 3 cm) could effectively stimulate the metamorphosis of *R. venosa* [13]. Additionally, a previous study indicated that oyster reefs can significantly promote the recovery of *R. venosa* resources [19], and the results of our field survey also showed that there were a large number of *R. venosa* individuals present around oyster reefs. Therefore, oyster reefs promote the recovery of *R. venosa* resources not only by providing sufficient food for the adults but also by stimulating the metamorphosis of the larvae.

However, oyster is also one of the most important economic species worldwide [20], and oyster reefs have significant ecological functions, promoting resource recovery and improving the aquatic environment [19]. Once the number of *R. venosa*, as a predator, exceeds a certain threshold, oyster resources will be destroyed [21], so it is of great significance to maintain the number of *R. venosa* individuals within an appropriate range for stabilizing the ecological balance of oyster reefs. Therefore, the metamorphosis mechanism of *R. venosa* induced by juvenile oysters needs to be characterized urgently. In a previous study, we found dramatic alterations in the metabolome and symbiotic microbiota for competent larvae of *R. venosa* responding to the induction of juvenile oysters, which further proved that the induction of juvenile oysters on the metamorphosis of *R. venosa* at the molecular level. However, the upstream regulatory mechanism of these changes in metabolites and microorganisms is still unclear.

Transcriptomics has been widely applied to investigate the mechanism underlying metamorphosis in invertebrates, including the mussel *M. sallei*, cnidarian *Nematostella vectensis*, pearl oyster *Pinctada fucata* and abalone *Haliotis diversicolor* [3], [22], [23], [24], revealing the critical pathway and genes regulating metamorphosis and providing novel insights into the metamorphosis mechanism in marine invertebrates. MicroRNAs (miRNAs) participate in RNA silencing and posttranscriptional gene expression regulation in eukaryotes [25], [26], providing further concrete support for conclusions drawn from the transcriptome; however, few studies have been conducted to investigate metamorphosis in marine invertebrates. Only Song et al. [27] have explored the alterations in miRNA expression that occur during metamorphosis in *R. venosa*, and most studies have been conducted to investigate the metamorphosis of insects and amphibians [28], [29]. With the rapid development of omics technology and increasing generation of genome data, the integrated analysis of miRNAs and mRNAs is convenient and effective for revealing the development mechanisms of animals, plants, and even fungi [30], [31], [32].

Herein, to further investigate the mechanism underlying the induction of juvenile oysters on the metamorphosis of *R. venosa*, we obtained the miRNA and mRNA expression profiles of competent larvae under induction of juvenile oysters for 2 h (Oe) and 12 h (Ol) and of those in the control treatment for 2 h (Ce) and 12 h (Cl) by sequencing on the Illumina NovaSeq 6000 platform. Then, we identified the differentially expressed miRNAs (DEMs) and mRNAs (DEGs), and the target genes of the DEMs and DEGs were subjected to Gene Ontology (GO) and Kyoto Encyclopedia of Genes and Genomes (KEGG) enrichment analyses. We further constructed the miRNA–mRNA regulatory network of the mechanism underlying the induction effect of juvenile oysters on the metamorphosis of *R. venosa*. These results will provide new insight into the metamorphosis mechanism of carnivorous gastropods and provide a theoretical basis for the protection and restoration of two important fishery resources and the maintenance of the steady state of the oyster reef ecological environment.

## 2 Materials and methods

### 2.1 Larval culture and sample collection

**Fig. 1:**
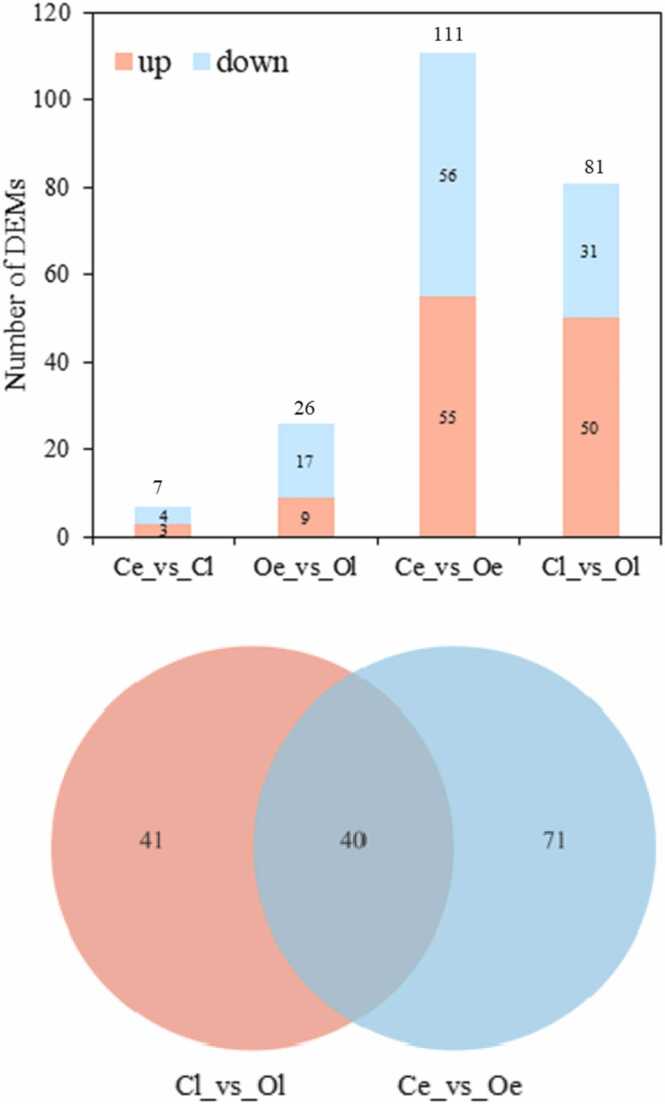
Statistics of differentially expressed miRNAs in the four groups (A) and Venn diagram of differentially expressed miRNAs in the Cl vs. Ol and Ce vs. Oe comparisons (B).

Culturing of *R. venosa* larvae and the sample collection were performed as described by Yang et al. [33], and the competent larvae (shell height>1200 µm) were used to conduct the juvenile oyster induction assays. The controls and treatments group were included in the assays, and the detailed experimental design is shown in Fig. 1. Four group samples were collected, including the control group (without juvenile oysters) at 2 h (Ce) and 12 h (Cl) and the oyster induction group (with juvenile oysters) at 2 h (Oe) and 12 h (Ol), and each sample contained 50 larvae. Nine samples were randomly collected from each pool at 2 h and 12 h after treatment and washed with PBS, total thirty-six samples were obtained which were stored at −80 °C for RNA extraction.

### 2.2 Library preparation, transcriptome sequencing and read mapping

Total RNA was extracted from the larval samples using TRIzol® Reagent according to the manufacturer’s instructions (Invitrogen), and the 1 % agarose gels were used to monitor the degradation and contamination of RNA, and the 2100 Bioanalyser (Agilent) and ND-2000 (NanoDrop Technologies) were used to determine the quality and quantity of RNA.

The miRNA library was generated using the NEBNext® Multiplex Small RNA Library Prep Set (Illumina, USA) with 3 μg of total RNA per sample. PCR amplification was performed using LongAmp Taq 2X Master Mix according to the manufacturer’s instructions. And the Agilent Bioanalyzer 2100 system was used to assessed library quality. The mRNA library was prepared using a TruSeqTM RNA Sample Preparation Kit (Illumina, USA), and the double-stranded cDNA was synthesized by SuperScript double-stranded cDNA synthesis kit (Invitrogen, USA).

The Illumina NovaSeq 6000 platform (*Majorbio*. Shanghai, China) was used to sequence the miRNA and mRNA libraries, and 50-bp single-end and 125-bp paired-end reads were generated and deposited in the Sequence Read Archive (ncbi.nlm.nih.gov/sra) under BioProject PRJNA855327 and PRJNA855363. The raw reads were trimmed and quality controlled by SeqPrep and Sickle with default parameters, and clean reads were separately aligned to the reference genome of *R. venosa* (unpublished data deposited in BioProject PRJNA855327) with orientation mode using HISAT2 software v2.2.1 (http://daehwankimlab.github.io/hisat-genotype/) [34]. The mapped reads of each sample were assembled by StringTie v2.2.0 (https://ccb.jhu.edu/software/stringtie/index.shtml?t=example) via a reference-based approach [35].

### 2.3 Analysis of DEMs

The identical sequences of sizes ranging from 18 to 32 nt were counted and eliminated from the initial data set, then used the BLAST search of the Rfam database v10.1 (http://rfam.sanger.ac.uk/) to remove non-miRNA sequences. The Bowtie v1.3.1 (http://bowtie-bio.sourceforge.net/index.shtml) and miRbase v21.0 (http://www.mirbase.org/) were used to annotate the chromosomal location and analyze the known miRNA expression profile, respectively. The TPM algorithm was used to normalize the expression levels of miRNAs in each sample and DESeq2 R package was used to identify the DEMs between samples, with the significance thresholds set to P-adjusted value< 0.05 and |log_2_^FC^|≥ 1 (the p-adjusted values were obtained from the false discovery rate (FDR) controlled by Benjamini and Hochberg's approach). Additionally, target genes of miRNA were predicted by miRanda and RNAhybrid.

### 2.4 Analysis of DEGs

Comparative transcriptomic analysis was conducted to identify the DEGs among group, and the TPM algorithm was used to normalize the expression level of each transcript. RSEM v1.3.3 (http://deweylab.biostat.wisc.edu/rsem/) was used to quantify gene abundances. Essentially, DEG analysis was performed using the DESeq2 R package with the significance thresholds set to P-adjusted value< 0.05 and |log_2_^FC^|≥ 1. Additionally, the GO and KEGG analyses, were used to identify the DEGs that were significantly enriched in GO terms and metabolic pathways with the significance enrichment threshold set to a P-adjusted value< 0.05 corrected by the Bonferroni method. Moreover, Gene Set Enrichment Analysis (GSEA) 57 v4.1.0 (http://www.gsea-msigdb.org/gsea/index.jsp) was performed to fully understand the gene expression trends of the gene sets identified in the KEGG or GO term analyses.

### 2.5 Integrated analysis of DEMs and DEGs

To further analyze the regulatory effect of critical DEMs and their target DEGs, Cytoscape v3.8.2 software was used to construct a DEM-DEG regulatory network. The intersection of “candidate target genes” and “DEGs” was referred to as differentially expressed target genes (t-DEGs). The Spearman coefficient was calculated according to the expression of DEMs and t-DEGs, and those with a coefficient<−0.6 and P value< 0.05 are shown in the DEM-DEG regulatory network.

### 2.6 Verification by quantitative RT-PCR

To validate the accuracy of the mRNA-Seq and miRNA profiling results, ten random DEMs and DEGs were selected for analysis. The cDNA for quantitative real-time PCR (qRT–PCR) was synthesized using the Prime ScriptTM RT Reagent Kit with gDNA Eraser (TaKaRa, Japan). The primers used in the mRNA qRT–PCR assay were designed using Primer 5, and 60 S ribosomal protein L28 (RL28) was selected as housekeeping gene to normalize the data [36]. For the miRNA qPCR assay, miRNA-specific forward and reverse primers were designed according to the stem–loop primer method (Table S1), and 5.8 S rRNA was selected as housekeeping gene to normalize the data [37]. The SYBR PrimeScript RT–PCR Kit II (TaKaRa, Japan) was used to quantify the expression levels. The relative expression levels of mRNAs and miRNAs were estimated by the 2 ^−ΔΔCT^ method. All data are presented as the means± SE (N = 3). Statistical significance was analyzed using SPSS v.19, with a P value< 0.05 considered significant.

## 3 Results

### 3.1 Overview of mRNA and miRNA sequencing

For small-RNA sequencing, 221.95 M raw reads were generated from the 12 samples in four groups (Ce, Cl, Oe and Ol), and each sample yielded greater than 14.17 M reads. After removing adaptors and low-quality reads, the number of clean reads in the samples ranged from 13,096,937 to 23,747,694 (Table S2). Then, the clean reads were used for the small-RNA distribution analysis, and result showed that the length of these small RNAs range 18–32 nucleotides (nt), and 22 nt length reads were most in each sample (Fig. S2). A total of 2220 miRNAs were obtained, comprising 379 known miRNAs and 1841 novel mRNAs.

There is approximately 95.54 Gb of clean reads were generated from transcriptome sequencing and filtering, and each cDNA library was greater than 6.73 Gb in size (Q30 >93.64 %), in which the efficiency of alignment compared with the reference genome ranged from 74.59 % to 77.18 % (Table S2). These results indicate that the sequences were of good quality for subsequent analysis. A total of 61454 transcripts were obtained, comprising 22,963 known transcripts and 38,491 novel transcripts. Gene function annotations showed that 23,953 genes had significant matches in the COG, GO, KEGG, KOG, Pfam, SwissProt, eggNOG, or NR databases (Table S3).

Hierarchical clustering analysis (HCA) of miRNA and mRNA showed that the Oe and Ol groups induced by oysters were clustered together, and the control groups Ce and Cl were clustered together (Fig S3 A, B). Additionally, principal component analysis (PCA) showed similar results: the groups induced by oysters were significantly separated from the control groups, while the separation of Ce and Cl and that of Oe and Ol were not significant (Fig S3 C, D). These results indicate that the induction of oysters could dramatically alter the miRNA and mRNA profiles, while the effect of the treatment time was minimal.

### 3.2 Expression analysis of DEMs

Differential expression analysis of the miRNAs among these four groups was performed according to the TPM value (Table S4). The DEMs in the Ce vs. Oe and Cl vs. Ol comparisons were regarded as those induced by juvenile oysters, while the DEMs in the Oe vs. Ol and Ce vs. Cl comparisons were regarded as those induced by the treatment time in the experiment. There were 81 DEMs in Oe vs. Ce and 111 in Ol vs. Cl, while there were only 7 in Ce vs. Cl and 26 in Oe vs. Ol. (Fig. 1A); so, the DEMs induced by treatment time in the experiment have been ignored. Forty DEMs were common to the Oe. vs. Ce and Ol vs. Cl comparisons (Fig. 1B). We further selected 38 DEMs whose TPM was> 10 in each sample to perform comparison analysis (Table 1). Additionally, 577 potential target genes were predicted according to the 38 DEMs. These DEMs and their potential target genes were considered core miRNAs and genes that respond to induction by juvenile oysters and may regulate the metamorphosis of *R. venosa*.

**Table 1:** Thirty-eight differentially expressed miRNAs that met the following criteria: average TPM> 10 (in twelve samples) and Padjust< 0.05 in the comparisons Cl vs**.** Ol and Ce vs. Oe.

**miRNA name**	**Ol**	**Oe**	**Cl**	**Ce**	**Log2FC** **(Ol/Cl)**	**Padjust**	**Log2FC** **(Oe/Ce)**	**Padjust**
bmo-miR-263a-5p	5268.776	2506.192	4388.492	2585.428	-1.291081953	4.13E-55	-0.87483854	0.000104
cel-miR-1–3p	1660.749	1449.722	1468.003	2084.331	-0.415200033	1.06E-05	0.407391912	0.041721
chr1_2090	1339.824	2502.395	1202.123	2551.304	0.682650087	3.23E-14	0.986139019	5.20E-13
chr1_636	730.573	612.7149	767.4971	547.3097	-0.473393379	1.06E-05	-0.59789735	0.01615
chr10_17992	11270.71	15128.57	11105.6	16446.41	0.205427825	0.036284	0.463833603	0.000445
chr13_22149	1466.489	955.5077	1362.225	913.4748	-0.836499792	1.71E-19	-0.68545849	0.00074
chr16_25439	11105.11	14906.14	10908.69	16222.86	0.205377557	0.036727	0.469921533	0.000388
chr2_4080	2123.337	1769.48	2439.768	1258.533	-0.481619837	1.21E-08	-1.06742457	2.33E-09
chr2_4084	1339.824	2502.395	1202.123	2551.304	0.682650087	3.23E-14	0.986139019	5.20E-13
chr23_34049	1407.388	2116.093	1444.106	2497.411	0.369086295	5.52E-05	0.68618473	7.43E-06
chr23_34050	1407.388	2116.093	1444.106	2497.411	0.369086295	5.52E-05	0.68618473	7.43E-06
chr23_34614	1407.388	2116.093	1444.106	2497.411	0.369086295	5.52E-05	0.68618473	7.43E-06
chr25_36630	3756.739	3116.248	4291.291	2935.169	-0.487685248	2.27E-11	-0.64431716	0.000493
chr27_39190	1690.12	1625.742	1884.827	1594.985	-0.274918209	0.026913	-0.3504792	0.04633
chr27_39191	282.4669	176.2865	306.0275	150.2018	-0.897996711	2.00E-10	-1.14061678	4.18E-07
chr31_42679	40.37067	16.8643	53.77973	13.91027	-1.474555889	0.00015	-2.06436777	4.91E-06
chr31_42687	5971.796	5279.981	6652.731	4542.981	-0.396432879	5.70E-07	-0.66088644	1.07E-05
chr31_42786	48.43287	120.9065	47.52503	111.8181	1.105837139	0.000293	1.126367763	0.0005
chr33_45116	123.6882	57.49593	152.817	75.3646	-1.324024391	6.79E-08	-1.14479775	0.001422
chr6_11346	116.8046	43.6158	124.96	37.69643	-1.63360046	4.35E-09	-1.85267358	1.25E-08
dre-miR-375	105.9198	47.00517	96.34087	45.96443	-1.39271271	2.18E-09	-1.19039134	0.000924
lgi-miR-133–3p	3747.987	6143.211	3575.197	5907.057	0.495197463	1.03E-09	0.622541529	5.37E-05
lgi-miR-137	393.3633	696.2842	511.6784	870.1319	0.603198861	3.14E-06	0.665853173	0.002726
lgi-miR-193	23116.4	42909.45	22675.59	41854.32	0.67441552	8.21E-20	0.783470785	7.48E-10
lgi-miR-1990	698.3893	492.8309	731.9296	518.5687	-0.720247223	8.10E-11	-0.6039075	0.002031
lgi-miR-1992	77.81427	269.6959	107.0823	243.1445	1.574142161	1.29E-14	1.100628128	0.002025
lgi-miR-1994a	284.5566	567.1138	284.456	500.5344	0.780521671	7.98E-07	0.711746508	2.67E-05
lgi-miR-252b	2590.401	1544.162	3207.817	1311.505	-0.964389217	4.80E-38	-1.41147239	3.90E-13
lgi-miR-263b	125025.9	86375.01	117898.1	82961.11	-0.752299439	4.65E-32	-0.62084357	0.005
lgi-miR-29	10345.81	5717.189	9347.004	6799.371	-1.073405924	1.23E-24	-0.55734334	0.000235
lgi-miR-2c	1307.198	2357.67	1383.686	2456.422	0.631917425	2.44E-14	0.731588538	0.000405
lgi-miR-31	4684.521	4184.945	5457.887	4492.356	-0.38070551	7.98E-06	-0.38194175	0.011048
lgi-miR-315	11941.86	28152.03	13347.71	25889.93	1.018389924	9.32E-27	0.863664001	3.92E-05
lgi-miR-375	37736.14	23167.51	39992.01	21384.18	-0.923571436	1.06E-35	-1.01833828	7.48E-10
lgi-miR-67	13534.96	23681.17	15710.55	25661.91	0.588933469	6.51E-21	0.61371073	0.001375
lgi-miR-745b	1048.555	881.5687	1021.735	766.5059	-0.467653125	5.58E-06	-0.51385111	0.01615
lgi-miR-750	33773.19	26106.88	34485.06	25225.46	-0.589979891	1.25E-18	-0.55523196	9.20E-06
lgi-miR-981	2065.73	3735.981	2363.12	3885.896	0.63697889	1.79E-15	0.609209834	3.49E-06

### 3.3 Expression analysis and functional enrichment of DEGs

**Fig. 2:**
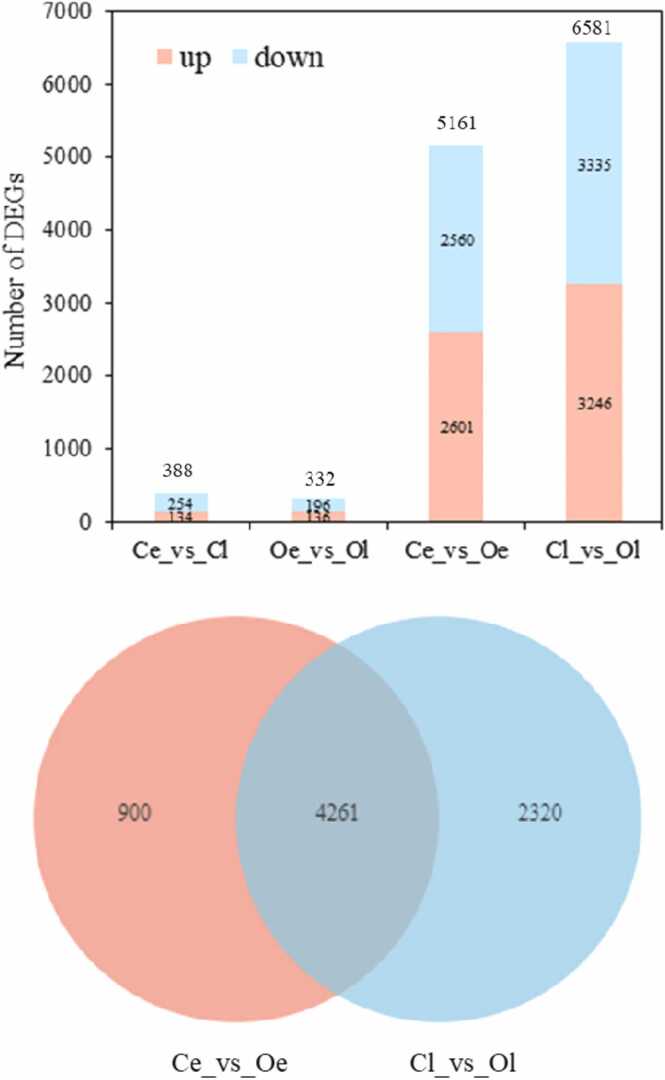
Statistics of differentially expressed genes in the four groups (A) and Venn diagram of differentially expressed genes in the Cl vs. Ol and Ce vs. Oe comparisons (B).

Differential expression analysis of the mRNAs among these four groups was also performed based on the TPM value for each identified mRNA (Table S5). The DEGs in the Ce vs. Oe and Cl vs. Ol comparisons were regarded as those induced by juvenile oysters, while the DEGs in the Ol vs. Oe and Cl vs. Ce comparisons were regarded as those induced by the treatment time in the experiment. There was a total of 5161 DEGs in Ce vs. Oe and 6581 DEGs in Cl vs. Ol, while there were only 388 DEGs in Ce vs. Cl and 332 DEGs in Oe vs. Ol (Fig. 2A). A total of 4261 DEGs were shared between Ce vs. Oe and Cl vs. Ol (Fig. 2B), which was considered core genes that respond to induction by juvenile oysters and may regulate the metamorphosis of *R. venosa*, while DEGs induced by treatment time has been ignored.

**Fig. 3:**
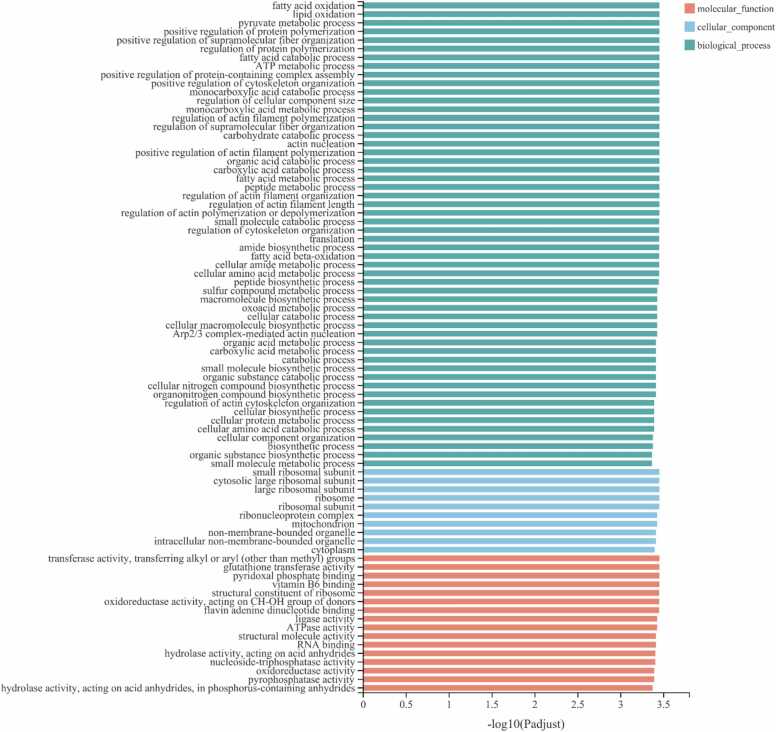
GO term enrichment of the 4261 differentially expressed genes common to the Cl vs. Ol and Ce vs. Oe comparisons (Padjust<0.05).

The results of functional enrichment analysis showed that these 4261 genes were significantly enriched in 289 GO terms, including 184 GO terms in the BP (biological process) category, 36 GO terms in the CC (cellular component) category and 69 GO terms in the MF (molecular function) category (P adjust<0.05). The top 80 GO terms are shown in Fig. 3; the results showed that fatty acid oxidation (GO:0019395), small ribosomal subunit (GO:0015935) and transferase activity, transferring alkyl or aryl (other than methyl) groups (GO:0016765) were the most significantly enriched GO terms in the BP category, CC category and MF category, respectively.

**Fig. 4:**
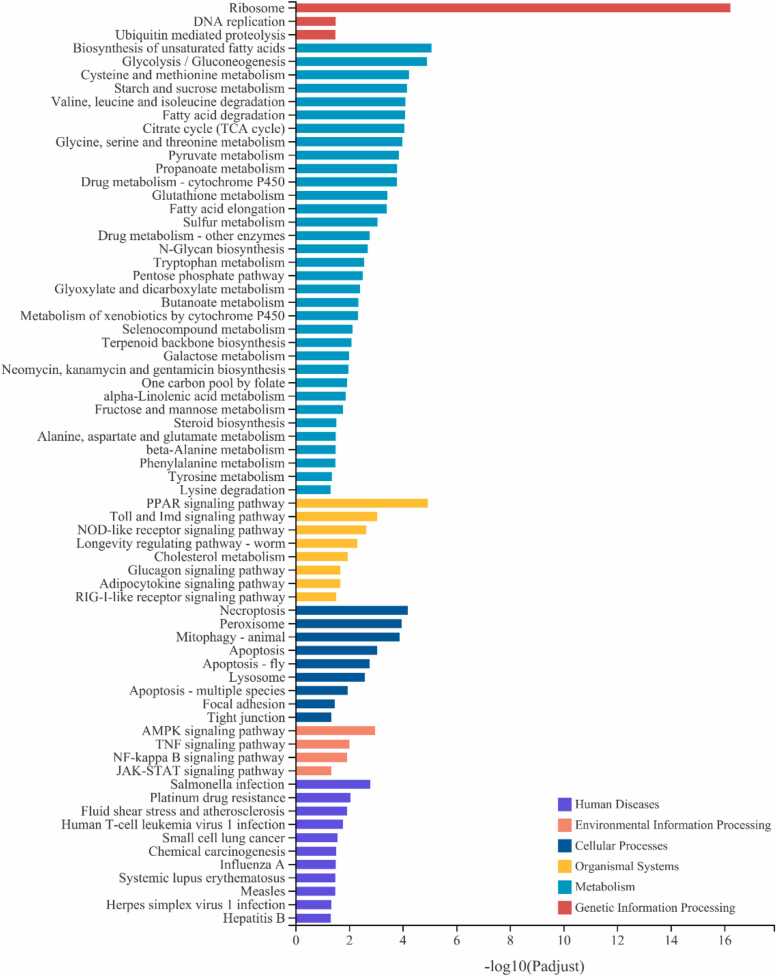
**KEGG enrichment of the 4261 differentially expressed gene**s common to the Cl vs. Ol and Ce vs. Oe comparisons (Padjust<0.05).

These 4261 genes were significantly enriched in 69 pathways (adjusted P < 0.05) (Fig. 4). For the KEGG enrichment analysis, 3 pathways were enriched in the genetic information processing category, and the ribosome was the most significantly enriched of these pathways. 35 pathways were enriched in the metabolism category, including the metabolism of fatty acids, amino acids and carbohydrates, and the most enriched of these pathways was biosynthesis of unsaturated fatty acids; 9 pathways were enriched in the cellular process category, most of which were related to necroptosis and apoptosis; 11 pathways were enriched in the human diseases category, and the most highly enriched of these pathways was Salmonella infection; 8 pathways were enriched in the organismal systems category, including the NOD-like receptor signaling pathway, PPAR signaling pathway, and Toll and Imd signaling pathway; 4 pathways were enriched in the environmental information processing category, including the AMPK signaling pathway, TNF signaling pathway, NF-kappa B signaling pathway, and JAK-STAT signaling pathway.

**Fig. 5:**
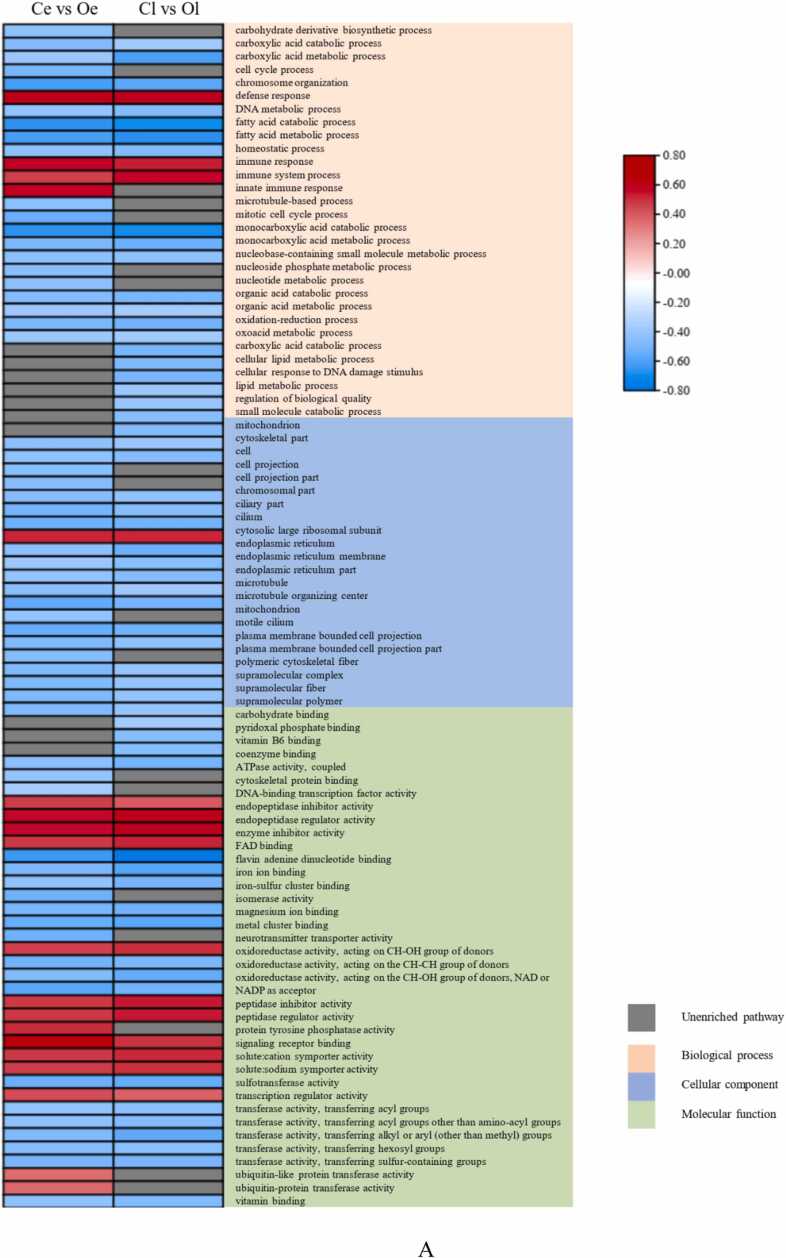

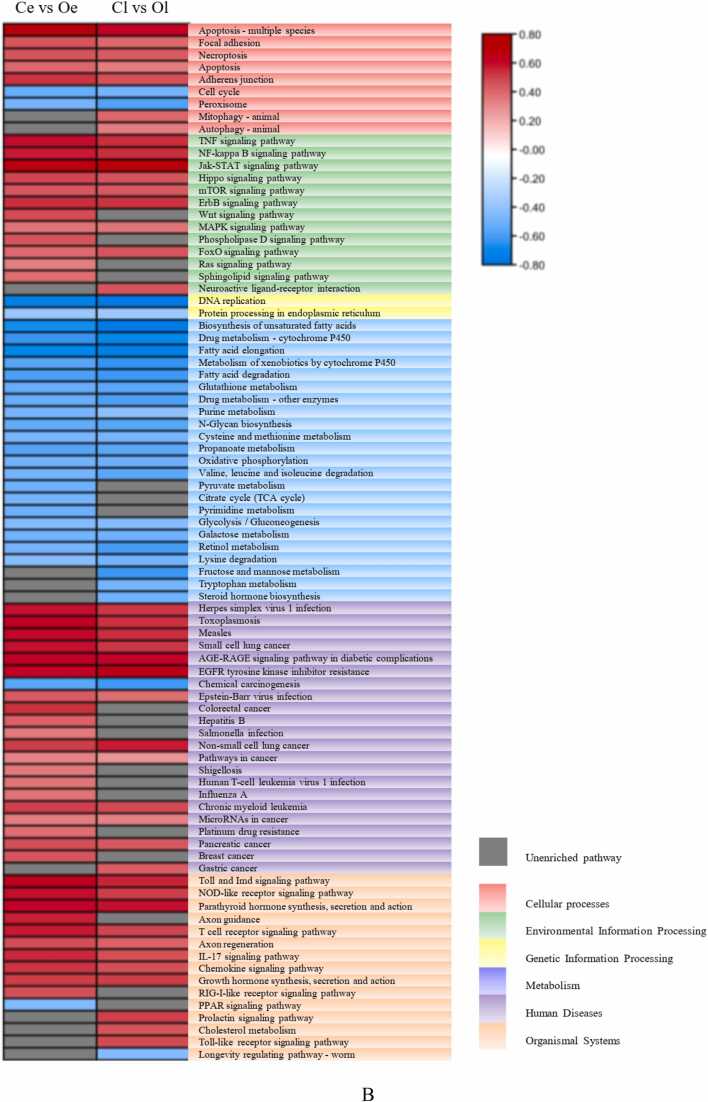
GSEA according to the GO (A) and KEGG (B) enrichment analyses of the Cl vs. Ol and Ce vs. Oe comparisons (Padjust<0.05).

The result of GSEA showed in Fig. 5, and enrichment scores (ESs) were used to draw a cluster heatmap for the Ce vs. Oe and Cl vs. Ol comparisons (P value<0.05). In total, the DEGs were enriched in 89 GO terms, including 37 GO terms in the MF category, 22 GO terms in the CC category and 30 GO terms in the BP category (Fig. 5A). The ESs of more than 79.78 % of the GO terms was less than 0, which means that most of the GO terms were depleted. In total, the DEGs were enriched in 84 pathways, including 9 pathways in the cellular processes category, 13 pathways in the environmental information processing category, 2 pathways in the genetic information processing category, 23 pathways in the metabolism category, 22 pathways in the human diseases category, and 15 pathways in the organismal systems category (Fig. 5B). The ESs of all the enriched pathways in the metabolism category were less than 0, while those of most of the enriched pathways in the other categories, especially in the environmental information processing category, were more than 0. This may mean that the pathways in the metabolism category were inhibited, while the pathways in environmental information processing and other categories were promoted.

Meanwhile, we further selected all of the genes related to digestive enzymes to analyze the changes involved in digestive system based on the TPM value, including the amylase, cellulase, protease and lipase. The results showed that the expression level of amylase and lipase have no significant changes, while cellulase and protease were dramatically decreased induced by juvenile oysters, which is not completely consistent with their changes before and after metamorphosis [14]. The decrease of both cellulase and protease would result from the starvation in the competent larvae under the induction of juvenile oyster.

### 3.4 Integrated analysis of DEGs and DEMs

**Fig. 6:**
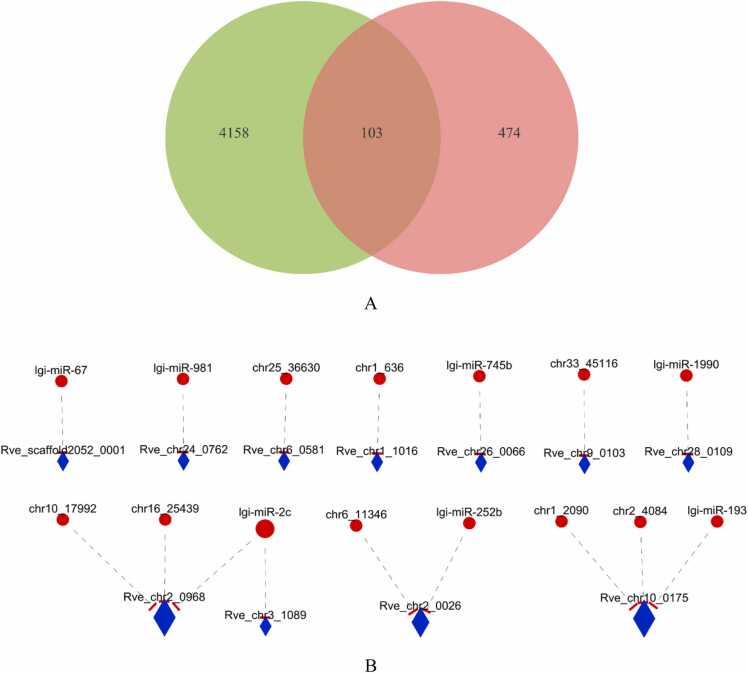
**Integrated analysis of differentially expressed miRNAs and genes. Venn diagram of “577 potential target genes of 38 core miRNAs” and “4261 core genes”; the intersection was referred to as differential expression target genes (t-DEGs) (A). Co-expression network among core mRNAs and t-DEGs based on the Spearman coefficient. Those with correlation coefficient**< −0.6 and *P* < 0.05 were selected and plotted; the miRNAs are represented by red dots, and t-DEGs are represented by blue diamonds (B).

Dramatic alterations in the miRNA and mRNA profiles were induced by juvenile oysters. To investigate the potential association between the miRNAs and mRNAs, the Spearman coefficient was calculated to construct the coexpression network of 38 DEMs and their t-DEGs. A total of 103 t-DEGs were obtained from the intersection of “candidate target genes of 38 DEMs” and “DEGs”, and we also selected the 27 t-DEGs whose TPM was> 10 to construct the coexpression network (Fig. 6A). The results showed that the coexpression network was composed of 15 DEMs and 11 t-DEGs (coefficient<−0.6 and P value<0.05) and showed 16 negative correlations between these DEMs and t-DEGs (Fig. 6B). In the coexpression network, there were 7 pairs of absolute one-to-one relationships, including lgi-miR-67 - Rve_scaffold2051_0001, lgi-miR-981 - Rve_chr24_0762, chr25_36630 - Rve_chr6_0581, chr1–636 - Rve_chr1_1016, lgi-miR-745b - Rve_chr26_0066, chr33_45116 - Rve_chr9_0103, and chr33–1990 - Rve_chr28_0109. There were 5 t-DEGs that were potentially regulated by more than one miRNA, including Rve_chr2_0026, Rve_chr10_0175, and Rve_chr2_0968. Only lgi-miR-2c had two target genes, Rve_chr2_0968 and Rve_chr3_1089, which may have more than one function in the response to induction by juvenile oysters and may regulate the metamorphosis of *R. venosa*. Additionally, the functional annotation of t-DEGs showed that Rve_chr24_0762 and Rve_chr28_0109 were involved in the NOD-like receptor signaling pathway (map04621) and ribosome (map03010), respectively, and Rve_chr3_1089 was involved in glycolysis/gluconeogenesis (map00010), the glucagon signaling pathway (map04922) and glycine, serine and threonine metabolism (map00260); these pathways were also enriched in the DEGs common to the Ce vs. Oe and Cl vs. Ol comparisons.

### 3.5 Quantitative RT-PCR validation of DEGs and DEMs

**Fig. 7:**
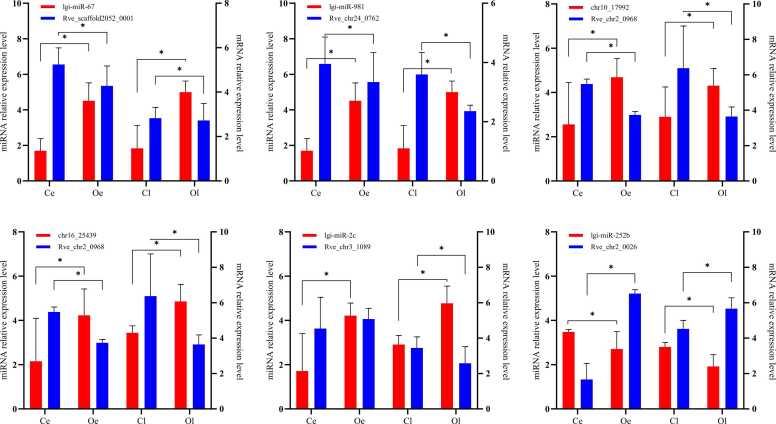
Validation of the DEMs and DEGs involved in different pairwise comparisons by qRT–PCR. 5.8 S and RL28 were selected to normalize the miRNA and gene expression levels, respectively. The data are shown as the means ( ± SEs) of three replicates, and ﹡ indicates a significant difference (P < 0.05).

The qRT-PCR analysis was performed on 6 key targeted miRNA-mRNA pairs to validate and identify the metamorphosis-related miRNAs in *R. venosa*, as shown in Table 2. The qRT-PCR results showed that the expression of miRNAs and mRNAs were consistent with the trend of high-throughput sequencing in the Ce vs. Oe and Cl vs. Ol comparisons (Fig. 7). For all the 6 pairs, there were inverse correlation between the expression levels of the miRNAs and mRNAs. Interestingly, the trends for all 6 pairs in Ce vs. Oe and Cl vs. Ol were almost the same, which means that the response to induction by oysters was fast and enduring.

**Table 2:** Coexpression of 16 DEM-target DEGs.

	**miRNA id**	**Average miRNA TPM**				**Target Gene id**	**Average mRNA TPM**				**Description**
		**Ce**	**Cl**	**Oe**	**Ol**		**Ce**	**Cl**	**Oe**	**Ol**	
1	lgi-miR-1990	698.3893	731.9296	492.8309	518.5687	Rve_chr28_0109	3199.357	2998.51	3814.053	3758.273333	60 S ribosomal protein L28-like
2	lgi-miR-67	13534.96	15710.55	23681.17	25661.91	Rve_scaffold2052_0001	52.66333	55.29667	27.04	24.463333	glutathione S-transferase-like
3	lgi-miR-745b	1048.555	1021.735	881.5687	766.5059	Rve_chr26_0066	137.8433	122.7633	251.75	276.116667	basic leucine zipper and W2 domain-containing protein 1-like
4	lgi-miR-981	2065.73	2363.12	3735.981	3885.896	Rve_chr24_0762	129.8933	139.1433	85.15	88.59	14–3–3 protein epsilon-like isoform X2
5	chr10_17992	11270.71	11105.6	15128.57	16446.41	Rve_chr2_0968	47.01	48.65667	29.16667	27.756667	protein chibby homolog 1-like
6	chr16_25439	11105.11	10908.69	14906.14	16222.86	Rve_chr2_0968	47.01	48.65667	29.16667	27.756667	protein chibby homolog 1-like
7	lgi-miR-2c	1307.198	1383.686	2357.67	2456.422	Rve_chr2_0968	47.01	48.65667	29.16667	27.756667	protein chibby homolog 1-like
8	lgi-miR-2c	1307.198	1383.686	2357.67	2456.422	Rve_chr3_1089	67.38667	70.71333	32.28	30.22	Probable phosphoglycerate mutase
9	chr25_36630	3756.739	4291.291	3116.248	2935.169	Rve_chr6_0581	30.32667	29.47	45.31	50.946667	eukaryotic peptide chain release factor subunit 1-like
10	chr1_636	730.573	767.4971	612.7149	547.3097	Rve_chr1_1016	10.88667	11.27667	13.91333	15.786667	GTP-binding protein 1-like
11	chr33_45116	123.6882	152.817	57.49593	75.3646	Rve_chr9_0103	65.08333	72.64667	105.5533	101.073333	
12	chr6_11346	116.8046	124.96	43.6158	37.69643	Rve_chr2_0026	512.8133	522.2867	804.3433	757.67	SARP-19 precursor
13	lgi-miR-252b	2590.401	3207.817	1544.162	1311.505	Rve_chr2_0026	512.8133	522.2867	804.3433	757.67	SARP-19 precursor
14	chr1_2090	1339.824	1202.123	2502.395	2551.304	Rve_chr10_0175	20.06333	21.24667	14.08667	16.1	
15	chr2_4084	1339.824	1202.123	2502.395	2551.304	Rve_chr10_0175	20.06333	21.24667	14.08667	16.1	
16	lgi-miR-193	23116.4	22675.59	42909.45	41854.32	Rve_chr10_0175	20.06333	21.24667	14.08667	16.1

## 4 Discussion

Metamorphosis, a key process in developmental biology, is an early evolutionary process involving the recognition of environmental signals and extensive morphological and physiological changes [7], [38]. Based on our previous studies about extensive changes that occur during metamorphosis in *R. venosa*, we carried out a further study to investigate the effect of the environmental signals from juvenile oysters on competent *R. venosa* larvae by integrated analysis of miRNA and mRNA profiles.

The recognition of environmental signals in metamorphosis usually involves the activation of signaling pathways. For *H. rufescens*, GABA in *Lithophyllum* sp. and *Lithothamnion* sp. induced metamorphosis by activating the cAMP-PKA pathway [5], and for *B. amphitrite*, larval settlement induced by an SIPC-containing extract of its adults activated the MAPK signaling pathway. In the present study, the DEGs were significantly enriched in the AMPK, TNF, NF-kappa B and JAK-STAT signaling pathways, and the GSEA results showed that almost all the pathways belonging to the environmental information processing category were promoted, which may further verify that induction by juvenile oysters activated the signaling pathway to stimulate the metamorphosis of *R. venosa*.

The AMPK signaling pathway has been found to be involved in the metamorphosis of *M. sallei* [3]. He et al. [3] indicated that the metamorphic inducer adenosine of *M. sallei* was transported into the cell via a nucleoside transporter and then catalytically acted upon by adenosine kinase (ADK), which resulted in the elevation of the (AMP + ADP)/ATP ratio and further activated the downstream AMPK-FoxO signaling pathway, inducing larval settlement and metamorphosis in *M. sallei*. Our previous study on the metabolite profile indicated that the level of AMP was increased under induction of oysters (Fig S4), and the present result also showed that the expression of the 5′-AMP-activated protein kinase subunit (AMPK) in DEGs was upregulated, as well as that of almost all the downstream genes of AMPK (Fig. 9), which suggested that the AMPK signaling pathway may be also activated during the response of competent larvae to induction by juvenile oysters.

**Fig. 8:**
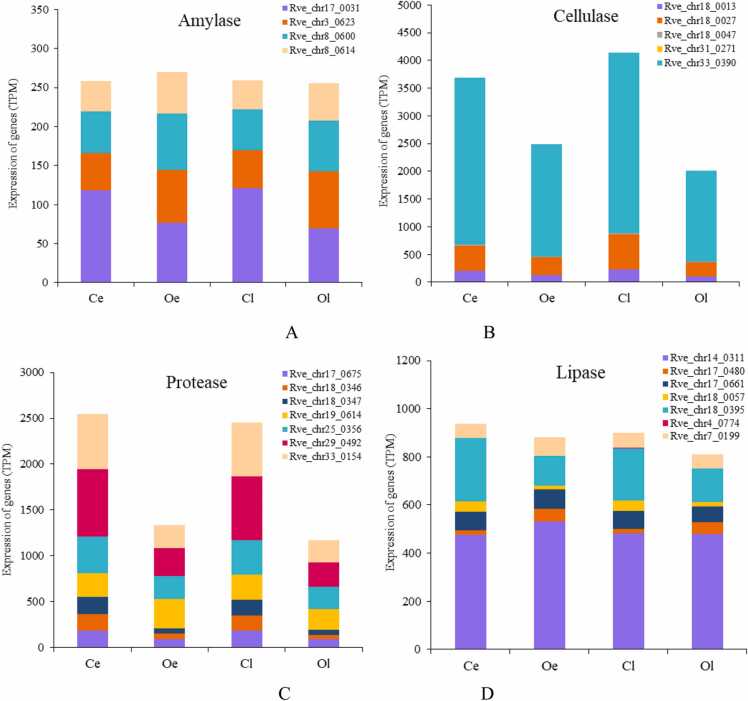
Expression of digestive enzymes: Amylase (A), Cellulase (B), Protease (C), Lipase (D).

AMPK, as a monitor of energy levels in cells, senses the AMP/ATP and ADP/ATP ratios, which play an important role in maintaining energy balance at both the cellular and individual levels [39]. During metamorphosis, mollusks usually feed nothing due to the degeneration of the velum under low-energy conditions [40], which was also found in the metamorphosis of *R. venosa*, and the decrease in the expression of cellulase and protease also indicated the starvation condition (Fig. 8). Our present results showed that the activation of the AMPK signaling pathway may lead to an increase in glycogen uptake and utilization and free fatty acid (FFA) oxidation, which could increase ATP levels, while inhibition of glycogen, fatty acid, protein and cholesterol synthesis could lead to ATP consumption; the results of GSEA were consistent with these findings. Li et al. (2013) also indicated that starvation conditions can activate the AMPK signaling pathway [41]. Additionally, the AMPK signaling pathway can also be activated by low cellular energy caused by environmental stress in aquatic animals. Dong and Zhang (2016) indicated that when the limpet *Cellana toreuma* responds to the low cellular energy status caused by high temperature and desiccation, AMP can induce the upregulation of AMPK and then affect carbohydrate and lipid metabolism to produce ATP for stress responses [42].

In addition to maintaining energy balance, the AMPK signaling pathway is activated to regulate the autophagy induced by starvation conditions [41], which helps to maintain cellular homeostasis by metabolizing nutrients [43]. During the development of animals, autophagy helps reshape tissues and organs by degrading and utilizing old tissues and cells and providing nutrients for the organism [44]. As a critical developmental process of marine invertebrates, metamorphosis is often accompanied by autography, which has been widely reported, such as *Ciona intestinalis* and *Crassostrea angulata* [45], [46]. In the present study, the level of mTOR, which is a regulator of autophagy and a downstream gene of AMPK, was significantly increased under the induction of juvenile oysters [47]. Additionally, the expression of a series of genes related to autophagy, including ATG1, ATG2, ATG4, ATG8, ATG9 and ATG18, which has been reported to be involved in the metamorphosis of *Bombyx mori*, *Drosophila melanogaster* and *Caenorhabditis elegans*, was significantly increased under the induction of juvenile oysters [48], [49], [50], [51]. These results suggested that the juvenile oysters induced autophagy in competent larvae of *R. venosa*, which may prepare them for metamorphosis, and the AMPK signaling pathway may play an important role in the induction of autophagy, but which need further confirm.

Previous studies have indicated that high level of autophagy can induce apoptosis via molecular switches, including Atg5, p53 and Bcl-2 [52]. Apoptosis occurs synergistically with autophagy during the metamorphosis of marine invertebrates, and autophagy is activated during metamorphosis to provide nutritional support for metamorphosis, whereas apoptosis, as the main driver, is required for remodeling organs and tissues during metamorphosis [53], [54]. We found that the TNF, NF-kB and JAK-STAT signaling pathways were significantly activated by juvenile oysters, which were closely related to apoptosis in marine invertebrates [55]. Additionally, Bax and Bak, caspase-3 (CASP3), caspase-3 (CASP7) and caspase-3 (CASP9) were upregulated in juvenile oysters, as well as the inhibitor of apoptosis (IAP) and Bcl-2, the levels of which were also significantly changed during the metamorphosis of *R. venosa* [18]. Previous studies have reported that the activation of Bax and Bak can change mitochondrial permeability, further activate CASP3 and CASP7, and induce apoptosis. However, overexpression of Bcl-2 can inhibit the change in mitochondrial permeability, and upregulation of IAP can inhibit the combination of CASP3 and CASP7 with their substrates [56], [57], [58]. Therefore, although induction by oysters activated apoptosis, the process was also strictly controlled to maintain balance in the development of *R. venosa.* Furthermore, we inferred that apoptosis was induced by high levels of autophagy on the one hand and, on the other hand, was activated by some cytokines that are affected by oyster induction.

As a critical developmental process, in addition to the degradation of old tissue and organs, the development of new tissue and organs must occur during metamorphosis. Previous studies have found that the competent larvae of *R. venosa* need to undergo the transition from herbivorous to carnivorous, and their digestive system changes dramatically, including digestive enzyme alternation [14], radula development [59], and the upregulation of the critical gene SARP-19, which has also been reported to be highly expressed in the gastropod larval digestive gland and sensitive to metamorphic inducers [60]. In the present study, the SARP-19 precursor was significantly increased under the induction of juvenile oysters, and the expression of chr6_11346 and lgi-miR-252b, which target the SARP-19 precursor, were both downregulated (Table 2). Both SARP-19 and its upstream miRNAs were sensitive to metamorphic inducers from juvenile oysters, which may suggest that the digestive system of competent larvae responds quickly to changes for completion of metamorphosis. Additionally, we found that the protein chibby homolog 1-like was downregulated (Table 2); this protein has been reported to be an antagonist of the Wnt/β-catenin signaling pathway [61]. And the Wnt/β-catenin signaling pathway is one of a few central signaling pathways that regulate many aspects of animal early development [62]. The lower expression of the protein chibby homolog 1-like gene in the groups after the induction of juvenile oysters (Ce and Cl) may promote the Wnt/β-catenin signaling pathway, then regulated the metamorphosis. This phenomenon is known as the metamorphosis delay, which can enhance the probability of seeking metamorphic inducers and the dispersal capability of competent larvae to increase gene flow and enhance individual vigor and is of great significance in biological evolution [63].

## 5 Conclusions

**Fig. 9:**
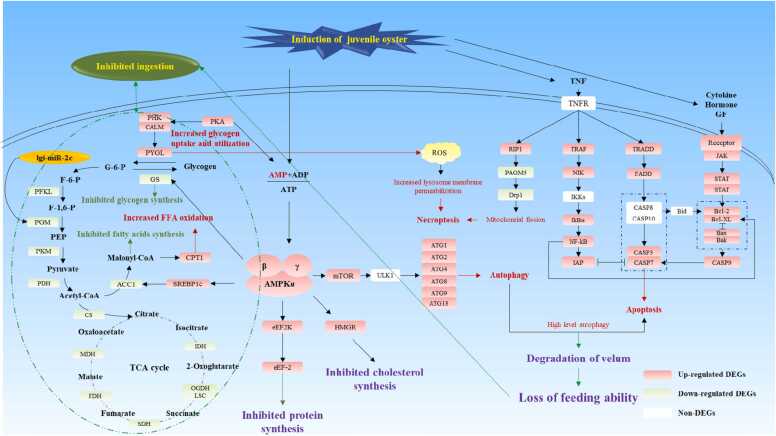
Schematic of the mechanism underlying the response of *Rapana venosa* to its metamorphic inducer, juvenile oysters, which is according to the result of KEGG analysis, and the pathways involved include map04152, map04668, map04630, map04064, map04210, map04214, map04215, map00010, map00020 and map01040 (All the genes were from the DEGs common to the two comparisons Cl vs. Ol and Ce vs. Oe, which have the same change trend) [3], [18], [38], [39], [40], [41], [42], [43], [44], [45], [46], [47], [48], [49], [50], [51], [52], [53], [54], [55], [56], [57], [58].

This study further revealed the response of *R. venosa* competent larvae to their metamorphic inducer, juvenile oysters, at the transcriptional level by integrated analysis of miRNA and mRNA profiles. Significant alterations in critical genes related to signal transduction, energy metabolism, autophagy and apoptosis, growth and development. According to these changes, we propose a hypothetical model of the response mechanism in *R. venosa* competent larvae to juvenile oysters, which may centered on the AMPK signaling pathway (Fig. 9). Induction of juvenile oysters increased the AMP level, which may activated the AMPK signaling pathway to maintained the energy balance by promoting the utilization and inhibiting the synthesis of organics and activating autophagy to deal with impending starvation caused by velum degeneration. The NF-kB and JAK-STAT signaling pathways were also activated by metamorphic inducers and regulated apoptosis, which may result in the degeneration of the velum. Additionally, the genes related to growth and development during metamorphosis also increased under the induction of juvenile oyster. However, which type of chemical cue is released from juvenile oysters and how the AMP level increases needs further investigation. Our results provide new insight into the mechanism of metamorphosis induced by metamorphic inducers in carnivorous gastropods, which may help to recovery the wild resource.

## CRediT authorship contribution statement

TZ conceived and designed the experiments. MY conducted the experiments. M-JY and HS analyzed the data. PS, JL, ZH, CZ, PH and ZY, contributed reagents, materials, and analytical tools. MY wrote the manuscript.

## Conflict of interest

The authors report no conflict of interest.
