# Supplementary material for: Integrated mRNA and miRNA transcriptomic analysis reveals the response of Rapana venosa to the metamorphic inducer (juvenile oysters)
Source: Comput Struct Biotechnol J. 2022 Dec 30;21:702–15. doi: 10.1016/j.csbj.2022.12.047 (PMC9826900; doi:10.1016/j.csbj.2022.12.047)
Supplement: Supplementary file 1 — Supplementary material. [file mmc1.docx]

**Table S1. Primers used in quantitative real-time PCR.**

| **Gene** | **Primer** | **Sequence (5’-3’)** |
| --- | --- | --- |
| Rve_scaffold2052_0001 | Rve_scaffold2052_0001-F | TGAGCAGCAGGAGGAGACCAAG |
|  | Rve_scaffold2052_0001-R | GCCAGGTCAACATAGGTCAGTCTG |
| Rve_chr24_0762 | Rve_chr24_0762-F | CAGGCTGAGCGATATGACGAGATG |
|  | Rve_chr24_0762-R | ACAACAGGTTGCGTTCCTCCAC |
| Rve_chr2_0968 | Rve_chr2_0968-F | AGGACAGGAGGTGGCCTTTGAG |
|  | Rve_chr2_0968-R | GCTGCTGGTTGGTCTTCTTCAGG |
| Rve_chr3_1089 | Rve_chr3_1089-F | CCCACCACCATTGAAACCTACTGAC |
|  | Rve_chr3_1089-R | ACACTCACAGGATGGAATGCACTTG |
| Rve_chr2_0026 | Rve_chr2_0026-F | TCTTCTCGTCTGCGTTGCTTTGG |
|  | Rve_chr2_0026-R | CTCGGTCTGCTGCTGATGGTTG |
| 60S ribosomal protein L28 | 60S ribosomal protein L28-F | CGTGCGTAACATCACCAAGA |
|  | 60S ribosomal protein L28-R | CACCACAGCTACCACACATT |
| lgi-miR-67 | lgi-miR-67-P | TCACAACCTGCATGAATGAGGAC |
| lgi-miR-981 | lgi-miR-981-P | TTCGTTGTCGACGAAACCTGCCT |
| chr10_17992 | chr10_17992-P | TATCACAGCCTGCTTGGATGAGC |
| chr16_25439 | chr16_25439-P | TATCACAGCCTGCTTGGATGAGC |
| lgi-miR-2c | lgi-miR-2c-P | TATCACAGCCAGCTTTGATGAGT |
| lgi-miR-252b | lgi-miR-252b-P | ATAAGTAGTGGTGCCGCAGGTA |
| 5.8s rRNA | 5.8s rRNA-P | TCGGCGAAGTTCTACCCATC |

**Table S2 Statistics of miRNA sequencing.**

| **Sample** | **Clean reads** | **Clean bases** | **Error rate (%)** | **Q20 (%)** | **Q30 (%)** | **GC content (%)** | **Useful reads**  **(18 nt-32 nt)** | **Total mapped** | **Multiple mapped** | **Uniquely mapped** |
| --- | --- | --- | --- | --- | --- | --- | --- | --- | --- | --- |
| Ce_1 | 20150967 | 4.94E+08 | 0.0236 | 98.51 | 95.63 | 47.84 | 18260694 | 39602159(77.18%) | 1750727(3.41%) | 37851432(73.77%) |
| Ce_2 | 23747694 | 5.77E+08 | 0.0233 | 98.69 | 95.99 | 47.91 | 22215381 | 44103882(76.83%) | 1836522(3.2%) | 42267360(73.63%) |
| Ce_3 | 18207530 | 4.35E+08 | 0.0235 | 98.61 | 95.78 | 48.25 | 16709617 | 42455066(76.71%) | 1764623(3.19%) | 40690443(73.52%) |
| Cl_1 | 17608765 | 4.04E+08 | 0.0236 | 98.51 | 95.61 | 49.33 | 16138851 | 43509548(76.32%) | 1885890(3.31%) | 41623658(73.01%) |
| Cl_2 | 13096937 | 3.11E+08 | 0.0234 | 98.65 | 95.77 | 47.81 | 11899952 | 41864575(76.43%) | 1712628(3.13%) | 40151947(73.3%) |
| Cl_3 | 16601175 | 3.89E+08 | 0.0233 | 98.68 | 95.99 | 48.22 | 15352059 | 40399716(76.37%) | 1665606(3.15%) | 38734110(73.22%) |
| Oe_1 | 14790595 | 3.58E+08 | 0.0238 | 98.42 | 95.42 | 47.76 | 13147833 | 43135083(75.89%) | 1849522(3.25%) | 41285561(72.64%) |
| Oe_2 | 19761851 | 4.6E+08 | 0.0231 | 98.78 | 96.11 | 47.61 | 18012019 | 43134739(75.54%) | 1875275(3.28%) | 41259464(72.25%) |
| Oe_3 | 15747763 | 3.75E+08 | 0.0232 | 98.69 | 96.01 | 47.72 | 14122235 | 38022324(75.88%) | 1600817(3.19%) | 36421507(72.68%) |
| Ol_1 | 17916846 | 4.13E+08 | 0.0235 | 98.55 | 95.78 | 47.73 | 15781399 | 41603601(74.59%) | 1644817(2.95%) | 39958784(71.64%) |
| Ol_2 | 16345716 | 3.95E+08 | 0.0232 | 98.72 | 96.01 | 47.06 | 14973245 | 39798666(75.91%) | 1657222(3.16%) | 38141444(72.75%) |
| Ol_3 | 15798666 | 3.82E+08 | 0.0235 | 98.61 | 95.64 | 47.36 | 14503891 | 34345906(76.0%) | 1381963(3.06%) | 32963943(72.94%) |

**Table S3 Statistics of gene sequencing.**

|  | **Expre_Gene number（percent）** | **Expre_Transcript number（percent）** | **All_Gene number（percent）** | **All_Transcript number（percent）** |
| --- | --- | --- | --- | --- |
| GO | 17427(0.4607) | 29632(0.4822) | 18342(0.4331) | 32339(0.4717) |
| KEGG | 13460(0.3558) | 23458(0.3817) | 14258(0.3367) | 25827(0.3767) |
| COG | 17128(0.4528) | 30153(0.4907) | 17790(0.4201) | 32795(0.4783) |
| NR | 22766(0.6018) | 38772(0.6309) | 24028(0.5674) | 42291(0.6168) |
| Swiss-Prot | 15785(0.4173) | 28071(0.4568) | 16335(0.3857) | 30541(0.4455) |
| Pfam | 18600(0.4917) | 32480(0.5285) | 19640(0.4638) | 35566(0.5187) |
| Total_anno | 23953(0.6332) | 40709(0.6624) | 25430(0.6005) | 44474(0.6487) |
| Total | 37830(1.0) | 61454(1.0) | 42348(1) | 68561(1) |

**Table S4 The expression level of each gene (partly).**

| Gene_id (TPM) | Ce_1 | Ce_2 | Ce_3 | Cl_1 | Cl_2 | Cl_3 | Oe_1 | Oe_2 | Oe_3 | Ol_1 | Ol_2 | Ol_3 |
| --- | --- | --- | --- | --- | --- | --- | --- | --- | --- | --- | --- | --- |
| Rve_chr10_0001 | 0 | 0.03 | 0 | 0 | 0 | 0 | 0 | 0 | 0 | 0 | 0 | 0 |
| Rve_chr10_0002 | 0 | 0 | 0 | 0 | 0 | 0 | 0 | 0 | 0 | 0 | 0 | 0 |
| Rve_chr10_0003 | 0 | 0 | 0 | 0 | 0 | 0 | 0 | 0 | 0 | 0 | 0 | 0 |
| Rve_chr10_0004 | 0 | 0 | 0.17 | 0 | 0 | 0 | 0 | 0 | 0 | 0 | 0 | 0 |
| Rve_chr10_0005 | 3.84 | 4.27 | 4.2 | 3.82 | 4.42 | 4.27 | 3.11 | 2.75 | 2.67 | 3.4 | 3.08 | 3.09 |
| Rve_chr10_0006 | 2.01 | 2.64 | 2.46 | 2.5 | 2.37 | 2.62 | 1.43 | 1.23 | 1.84 | 1.8 | 1.04 | 1.48 |
| Rve_chr10_0007 | 5.16 | 4.78 | 6.54 | 6.97 | 6.42 | 6.31 | 6.4 | 7.97 | 6.19 | 4.91 | 5.89 | 6.59 |
| Rve_chr10_0008 | 4 | 3.03 | 3.1 | 3.56 | 3.8 | 4.12 | 4.4 | 4.08 | 5.03 | 3.84 | 3.1 | 4.18 |
| Rve_chr10_0009 | 2.98 | 2.39 | 1.83 | 2.74 | 2.31 | 2.05 | 0.64 | 1.64 | 0.93 | 1.55 | 0.39 | 1.28 |
| Rve_chr10_0010 | 6.17 | 5.02 | 5.04 | 5.2 | 4.36 | 5.38 | 4.39 | 4.34 | 5.52 | 5.26 | 5.08 | 5.21 |
| Rve_chr10_0011 | 0.49 | 0.51 | 0.05 | 0.35 | 0.38 | 0.45 | 0.84 | 0.48 | 0.29 | 0.27 | 0.55 | 0.26 |
| Rve_chr10_0012 | 1.13 | 1.33 | 0.91 | 0.98 | 0.95 | 1.37 | 1.38 | 1.24 | 1.25 | 1.37 | 1.14 | 1.37 |
| Rve_chr10_0013 | 0 | 0 | 0 | 0 | 0 | 0 | 0 | 0 | 0 | 0 | 0 | 0 |
| Rve_chr10_0014 | 12.72 | 20.17 | 14.56 | 20.53 | 18.82 | 15.99 | 13.22 | 29.83 | 33.59 | 12.76 | 20.37 | 14.95 |
| Rve_chr10_0015 | 0.47 | 0.79 | 0.48 | 0.83 | 0.85 | 1.03 | 1.29 | 1.02 | 0.9 | 1.19 | 1.32 | 1.2 |
| Rve_chr10_0016 | 12.48 | 18.7 | 7.78 | 10.4 | 17.11 | 10.26 | 10.69 | 10.75 | 12.57 | 15.84 | 15.99 | 10.7 |
| Rve_chr10_0017 | 5.25 | 2.97 | 3.05 | 4.38 | 4.1 | 4.42 | 6.81 | 9.39 | 9.6 | 7.44 | 7.04 | 6.37 |
| Rve_chr10_0018 | 4.65 | 2.51 | 3.6 | 2.87 | 4.71 | 4.66 | 6.98 | 10.58 | 8.21 | 8.97 | 5.81 | 4.7 |
| Rve_chr10_0019 | 0.65 | 0.61 | 1.41 | 0.78 | 0.21 | 1.12 | 1.58 | 0.59 | 0 | 2.42 | 1.71 | 0.54 |
| Rve_chr10_0020 | 427.26 | 463.71 | 348.51 | 436.22 | 378.9 | 386.57 | 197.79 | 184.08 | 134.38 | 183.82 | 195.83 | 156.03 |
| Rve_chr10_0021 | 1.59 | 1.78 | 2.8 | 2 | 1.85 | 2.46 | 2.41 | 1.16 | 2.16 | 2.8 | 2.25 | 2.48 |
| Rve_chr10_0022 | 0.8 | 0.37 | 0.73 | 0.7 | 1.15 | 0.41 | 0.36 | 0 | 1.43 | 0.35 | 0 | 0.53 |
| Rve_chr10_0023 | 1.95 | 2.33 | 2.27 | 1.63 | 1.92 | 2.55 | 1.89 | 2.34 | 2.1 | 2.24 | 2.85 | 2.42 |
| Rve_chr10_0024 | 0.06 | 0.06 | 0 | 0 | 0.19 | 0 | 0 | 0 | 0 | 0 | 0 | 0 |
| Rve_chr10_0025 | 0 | 0 | 0 | 0 | 0 | 0 | 0 | 0 | 0 | 0 | 0 | 0.88 |
| Rve_chr10_0026 | 4.87 | 3.53 | 3.71 | 7.53 | 6.49 | 5.1 | 5.07 | 4.76 | 6.57 | 5.53 | 5.44 | 8.1 |
| Rve_chr10_0027 | 0 | 0 | 0 | 0 | 0 | 0 | 0 | 0 | 0 | 0.1 | 0 | 0 |
| Rve_chr10_0028 | 141.33 | 140.36 | 139.43 | 121.56 | 125.29 | 127.06 | 71.55 | 78.4 | 74.28 | 80.89 | 70.79 | 63.98 |
| Rve_chr10_0029 | 4.56 | 2.84 | 4.84 | 1.74 | 2.96 | 2.25 | 6.11 | 3.95 | 6.91 | 5.64 | 2.14 | 3.53 |
| Rve_chr10_0030 | 0 | 0 | 0 | 0 | 0 | 0 | 0 | 0 | 0 | 0 | 0 | 0 |

**Table S5 The expression level of each miRNA (partly).**

| miRNA name | Ol_3 | Ol_2 | Ol_1 | Oe_3 | Oe_2 | Oe_1 | Cl_3 | Cl_2 | Cl_1 | Ce_3 | Ce_2 | Ce_1 |
| --- | --- | --- | --- | --- | --- | --- | --- | --- | --- | --- | --- | --- |
| bmo-let-7-3p | 0 | 0 | 0 | 0 | 0 | 0 | 0 | 0.1577 | 0 | 0 | 0 | 0 |
| bmo-miR-1175-3p | 0.12 | 0 | 0.1106 | 1.219 | 1.2498 | 0.8661 | 0 | 3.626 | 0 | 0.2182 | 0.3382 | 0 |
| bmo-miR-184-3p | 5.641 | 7.8375 | 2.1007 | 5.3638 | 1.9164 | 3.4645 | 0.5926 | 0.9459 | 1.2373 | 12.8732 | 2.6213 | 10.9557 |
| bmo-miR-252-5p | 0 | 0 | 0 | 0.1219 | 0 | 0 | 0 | 0 | 0 | 0 | 0 | 0 |
| bmo-miR-263a-5p | 2931.624 | 2635.638 | 2189.022 | 2708.827 | 2277.434 | 2532.313 | 4015.284 | 3909.162 | 5241.03 | 5038.752 | 5466.071 | 5301.506 |
| bmo-miR-263b-5p | 0.12 | 0.3359 | 0.3317 | 0.6095 | 0.5833 | 0.7424 | 0.4741 | 5.5179 | 1.0123 | 0.4364 | 1.6911 | 0.8427 |
| bmo-miR-2780c | 0 | 0 | 0 | 0 | 0 | 0.2475 | 0 | 0 | 0 | 0 | 0 | 0 |
| bmo-miR-279a | 269.4463 | 230.8703 | 305.5941 | 308.5388 | 319.2891 | 296.9585 | 385.2074 | 356.2956 | 324.4008 | 312.8833 | 315.3958 | 304.4421 |
| bmo-miR-279c-3p | 0 | 0 | 0.2211 | 0 | 0 | 0.2475 | 0 | 0 | 0.1125 | 0 | 0 | 0 |
| bmo-miR-281-3p | 12.7222 | 11.0845 | 17.1372 | 15.7256 | 16.4144 | 11.7546 | 11.8525 | 11.5087 | 11.0233 | 5.3456 | 11.246 | 9.2702 |
| bmo-miR-283-5p | 0 | 0.5598 | 0 | 0 | 0 | 0.4949 | 0 | 0 | 0 | 0 | 0 | 0 |
| bmo-miR-285 | 0 | 0 | 0.1106 | 0 | 0.0833 | 0 | 0 | 0 | 0 | 0.2182 | 0 | 0 |
| bmo-miR-2a-3p | 11.642 | 12.7639 | 18.5745 | 14.8723 | 14.248 | 15.0954 | 22.9939 | 35.4719 | 3.3745 | 8.5094 | 1.2683 | 9.4809 |
| bmo-miR-2b-3p | 0 | 0 | 0.1106 | 0 | 0 | 0 | 0 | 0 | 0 | 0 | 0 | 0 |
| bmo-miR-316-5p | 0 | 0 | 0.7739 | 0 | 0 | 0.9899 | 0 | 0 | 0 | 0 | 0 | 0 |
| bmo-miR-317-3p | 0.8401 | 0.3359 | 0.9951 | 0.2438 | 0.4166 | 1.2373 | 0.1185 | 4.2566 | 0.6749 | 0.6546 | 0.4228 | 0.316 |
| bmo-miR-7-5p | 0 | 0.2239 | 0.6634 | 0 | 0.8332 | 0.1237 | 0 | 0.1577 | 0 | 0 | 0 | 1.2641 |
| bmo-miR-71-5p | 382.3857 | 219.5619 | 2227.166 | 1019.726 | 334.037 | 388.1495 | 645.7262 | 992.1098 | 1113.244 | 283.4278 | 507.8464 | 407.5731 |
| bmo-miR-87 | 50.5287 | 53.5189 | 41.7925 | 47.1768 | 41.8275 | 40.8318 | 28.5646 | 20.0219 | 45.893 | 72.5479 | 22.8303 | 44.5602 |
| bmo-miR-92a | 0 | 0 | 0 | 0.2438 | 0.0833 | 0.1237 | 0 | 0 | 0 | 0 | 0 | 0 |
| bmo-miR-9a-5p | 0 | 0 | 0.3317 | 0 | 0 | 0.2475 | 0 | 0 | 0 | 0 | 0 | 0 |
| bmo-miR-9b-5p | 0 | 0 | 0 | 0.1219 | 0 | 0.6187 | 0 | 0 | 0 | 0 | 0 | 0 |
| cel-let-7-5p | 79.0936 | 76.9194 | 76.5091 | 53.6377 | 51.5762 | 46.3998 | 47.0546 | 77.8805 | 101.2346 | 67.7477 | 60.5425 | 44.1388 |
| cel-miR-1-3p | 2054.153 | 2314.749 | 1884.092 | 1573.901 | 1449.716 | 1325.548 | 1540.83 | 1557.926 | 1305.252 | 1706.24 | 1649.613 | 1626.395 |
| cel-miR-124-3p | 0 | 0 | 0 | 0.1219 | 1.0832 | 0 | 0 | 0 | 0 | 0 | 0 | 0 |
| cel-miR-2-3p | 5.2809 | 5.1504 | 6.1915 | 6.5828 | 5.166 | 5.8154 | 3.3187 | 6.1485 | 2.6996 | 1.9637 | 3.7205 | 1.6855 |
| cel-miR-228-5p | 0 | 0 | 0 | 0 | 0.0833 | 0.1237 | 0 | 0 | 0 | 0 | 0 | 0 |
| cel-miR-252-5p | 0.12 | 0.112 | 0.3317 | 0.4876 | 0.0833 | 0.1237 | 0.1185 | 2.2071 | 1.0123 | 0 | 0.2537 | 0.2107 |
| cel-miR-34-5p | 18.0031 | 21.0493 | 18.3533 | 19.7484 | 20.4138 | 20.4159 | 15.7639 | 9.9321 | 8.9986 | 9.9276 | 11.246 | 16.4336 |
| cel-miR-46-3p | 0 | 0 | 0 | 0 | 0 | 0 | 0 | 0 | 0 | 0.1091 | 0 | 0 |


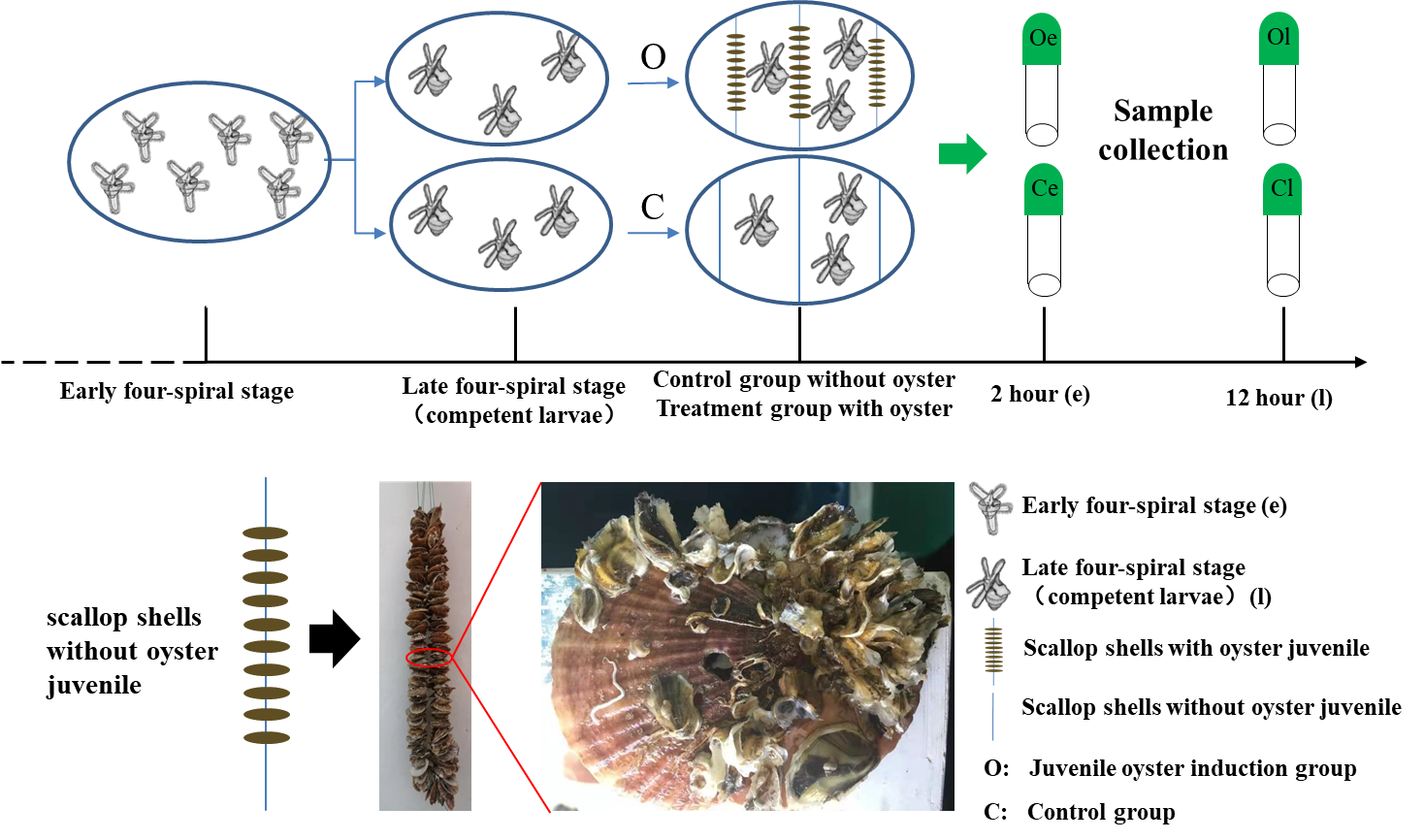


**Figure S1 Experimental design. The following controls and treatments were included in the assays: (1) seawater with scallop shells without juvenile oysters (control group, C) and (2) seawater with juvenile scallop shells with juvenile oysters (juvenile oyster induction group, O). collected samples from two pools at 2 hours (early stage, e) and 12 hours (later stage, l), and divided into four sample groups, including the control group at 2 hours (Ce) and 12 hours (Cl) and the treatment group at 2 hours (Oe) and 12 hours (Ol).**


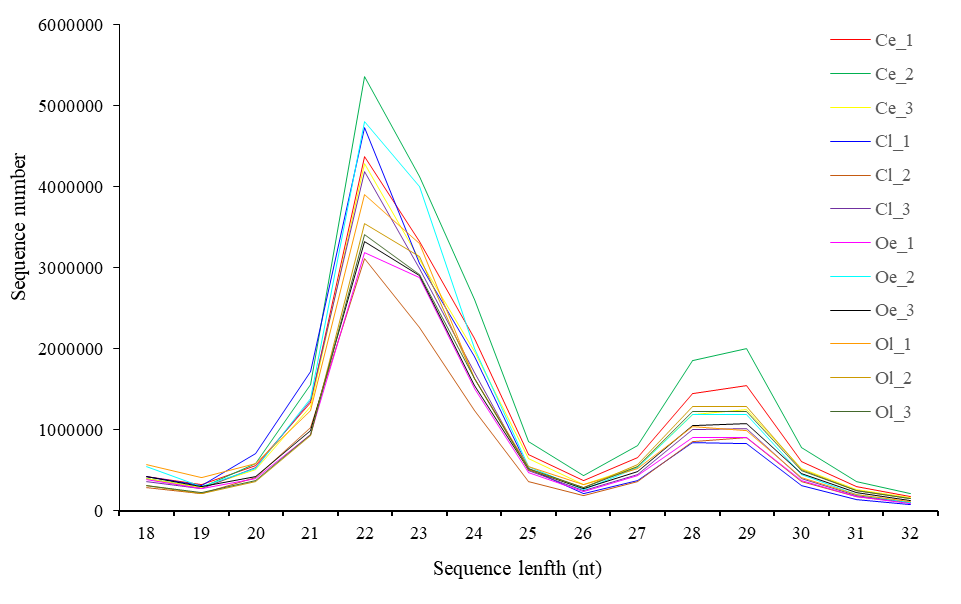


**Figure S2 Length distribution and frequency of miRNAs.**


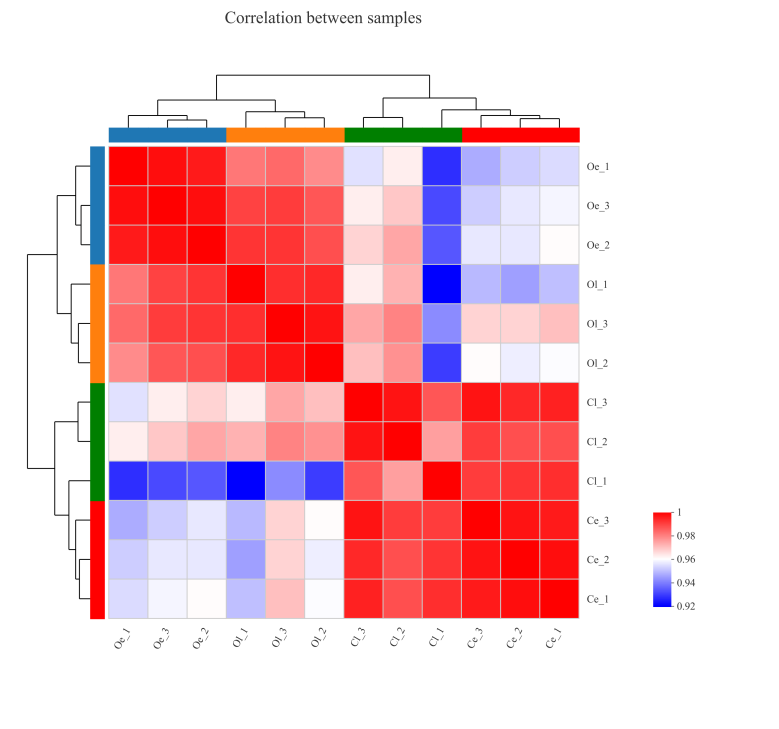
**
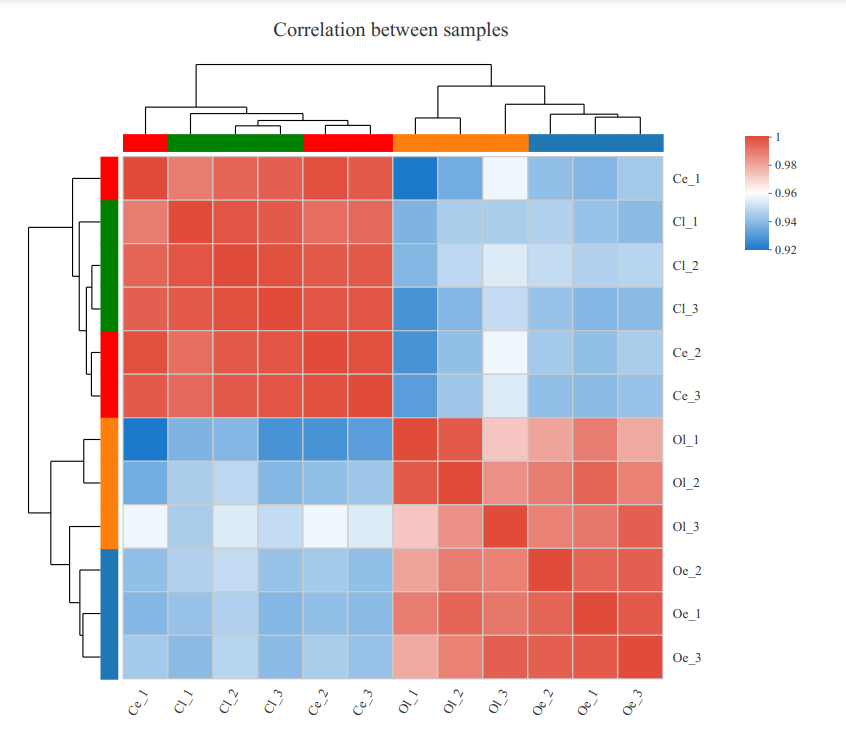
**

**A B**


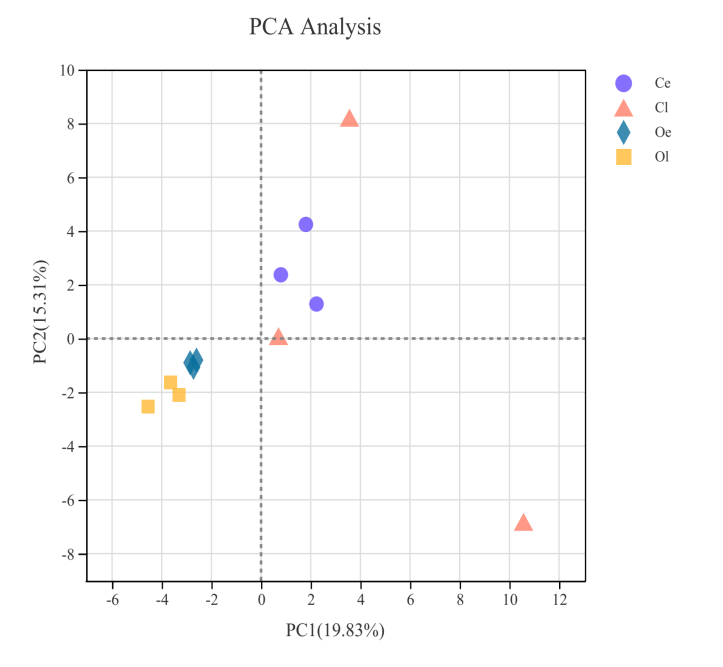

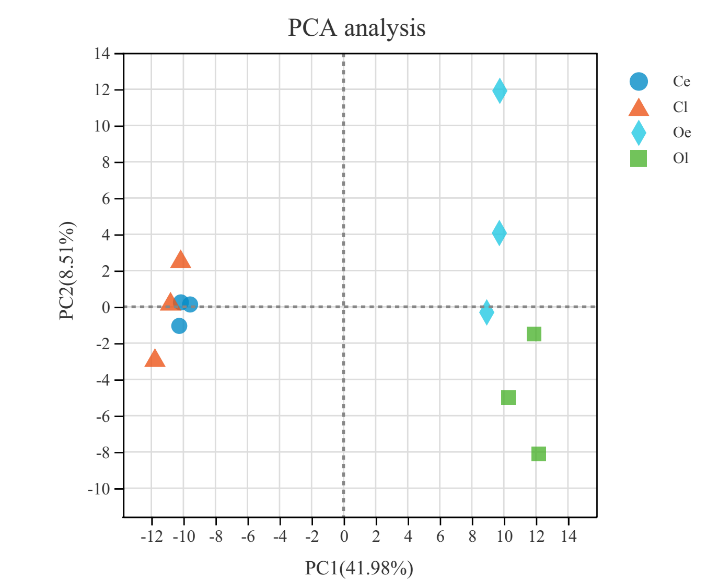


**C D**

**Figure S3 Relationships between samples. Hierarchical clustering analysis (HCA) of miRNA (A) and mRNA (B) profiles and principal component analysis (PCA) of miRNA (C) and mRNA (D) profiles.**


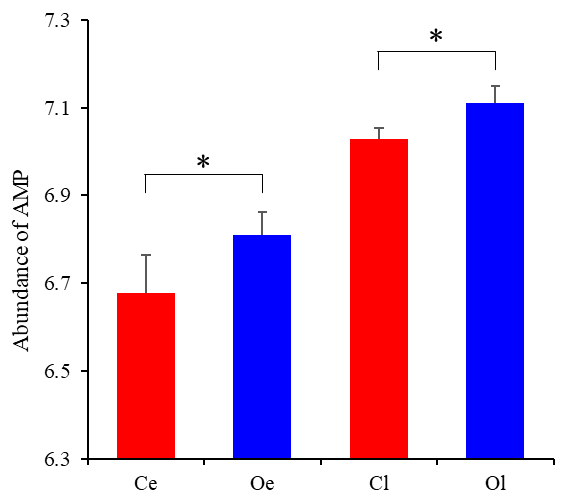


**Figure S4 Variation in AMP abundance in competent larvae of *Rapana venosa* induced by juvenile oysters.**
